# The Bristol Royal Infirmary 1904-74

**Published:** 1981

**Authors:** 


### Book Review

**THE BRISTOL ROYAL INFIRMARY 1904-1974**
**C. Bruce Perry**
**Portishead Press**
**£4.95**

Ostensibly this book takes up the story of the Bristol Royal Infirmary in 1904, the year of Sir George White's appointment as President and treasurer, an account of which ends the monumental History of the Bristol Royal Infirmary by Munro Smith. Actually Professor Perry deals in the first chapter with the early years in some detail and the book is in reality a concise history of the Bristol Royal Infirmary from its foundation in 1736 to 1974 and the reorganisation of the National Health Service.

Though little over 100 pages in length there is a wealth of biographical detail and chapter two begins with a vignette of Sir George White, that great Edwardian entrepreneur who developed the Bristol Tramways and founded the Bristol Aeroplane Company. It was due to his efforts that the King Edward VII Memorial Building got off the ground to be opened by King George V in 1912. A theme constantly running through the book is the need for ever more funds to relieve the ever increasing debt, and the part played by the great benefactors notably H.H. Wills, and by many fund raising schemes such as the Bristol Medical Institutions Contributory Scheme. In 1921 things were so bad that nearly 100 beds had to be closed down. Another theme is the protracted effort to achieve the amalgamation of the Royal Infirmary and the General Hospital begun in 1919 and only fully achieved in 1940.

In the era following the Second World War the impact of the National Health Service is fully dealt with and the reasons for developing the St. Michael's Hill site rather than the wholesale move to Ashton Park – a superficially attractive scheme ardently advocated are discussed.

A chapter is devoted to the development of specialisation and another to relations with the University and the Medical School. Throughout the story frequent reference is made to individual members of the Medical Staff and another chapter is devoted to the achievements and distinctions of other staff members who had escaped mention.

Another most interesting chapter deals with the Nursing Services and the gradual liberation during this period of the Nurse from almost total bondage as well as the steady improvement in training and nursing standards.

All the important facts are there but we are treated also to titbits – for example – the day that Matron discovered 2 bear cubs in the Children's Ward, smuggled in from the Zoo by Dr. Richard Clarke as cases of pneumonia and tucked up inside a steam tent – and the Bristol nurse working in a War Emergency Hospital in St. Petersburg who has a disagreement with Rasputin and ordered him out of the hospital, not only did she survive this encounter but lived to the age of 92.

Tables of different conditions treated show the changing incidence of diseases, some of them predictable others less so. Space is found for an episode not entirely to Bristol's credit about which many of us have heard rumours but now are given the facts. In 1927 Dr. Todd described the relief of inoperable cancer with lead selenide, great interest was aroused nationally and internationally, a successful appeal produced large sums of money. A laboratory was instituted and beds provided. With hindsight we know that the claims made were not substantiated but the work continued until 1940 when the outbreak of the War demanded a reappraisal. The problem of how to wind up research that is useful only to the researchers may still be with us.

As Professor of Medicine from 1935-39 the author has himself been at the centre of affairs for half the period he relates and he is uniquely qualified to tell this story. Bristol is greatly in his debt, and not only Bristol because this is a microcosm of the national scene. We also rejoice that he has been more fortunate than Munro Smith who did not live to see the publication of his great book.

To anyone who has lived and worked in the Bristol Medical scene this book is of absorbing interest and to the reviewer an occasion for no little shame in finding himself ignorant in so many important matters. This book should find its way into the hands of every graduate of the Bristol Medical School.

